# Dynamic changes of gut fungal community in horse at different health states

**DOI:** 10.3389/fvets.2022.1047412

**Published:** 2022-10-28

**Authors:** Yanfang Lan, Yaonan Li, Gang Yu, Zhengyi Zhang, Irfan Irshad

**Affiliations:** ^1^School of Physical Education and International Equestrianism, Wuhan Business University, Wuhan, China; ^2^Pathobiology Section, Institute of Continuing Education and Extension, University of Veterinary and Animal Sciences, Lahore, Pakistan

**Keywords:** horse, gut microbiota, fungal, diarrhea, amplicon sequencing

## Abstract

Accumulating studies indicated that gut microbial changes played key roles in the progression of multiple diseases, which seriously threaten the host health. Gut microbial dysbiosis is closely associated with the development of diarrhea, but gut microbial composition and variability in diarrheic horses have not been well characterized. Here, we investigated gut fungal compositions and changes in healthy and diarrheic horses using amplicon sequencing. Results indicated that the alpha and beta diversities of gut fungal community in diarrheal horses changed significantly, accompanied by distinct changes in taxonomic compositions. The types of main fungal phyla (*Neocallimastigomycota, Ascomycota*, and *Basidiomycota*) in healthy and diarrheal horses were same but different in relative abundances. However, the species and abundances of dominant fungal genera in diarrheal horses changed significantly compared with healthy horses. Results of Metastats analysis indicated that all differential fungal phyla (*Blastocladiomycota, Kickxellomycota, Rozellomycota, Ascomycota, Basidiomycota, Chytridiomycota, Mortierellomycota, Neocallimastigomycota, Glomeromycota*, and *Olpidiomycota*) showed a decreasing trend during diarrhea. Moreover, a total of 175 differential fungal genera were identified for the gut fungal community between healthy and diarrheal horses, where 4 fungal genera increased significantly, 171 bacterial genera decreased dramatically during diarrhea. Among these decreased bacteria, 74 fungal genera even completely disappeared from the intestine. Moreover, this is the first comparative analysis of equine gut fungal community in different health states, which is beneficial to understand the important role of gut fungal community in equine health.

## Introduction

As a forgotten or hidden organ, the role of the gut microbiota is increasingly recognized (1, 2). Studies showed that there are more than 100 trillion of microorganisms in the intestine including bacteria, fungi and protists, approximately 10 times the total number of host cells (3–5). Gut microbiota can not only synthesize nutrients such as amino acids, vitamins, and short-chain fatty acids required for host growth, but also play key roles in digestion, absorption and metabolism (6, 7). Additionally, gut microbiota is also involved in the construction of the intestinal barrier and the maturation of the immune system, suggesting its vital role in disease resistance (8, 9). However, the gut microbial homeostasis is easily disrupted by several host- and environmental-related factors (10, 11). The execution of intestinal functions depends on the normal gut microbial structure, whereas gut microbial dysbiosis may cause aetiopathologic consequences (12, 13). For instance, it has been demonstrated that gut microbial dysbiosis is an important driver of diarrhea (14, 15). Moreover, disrupted gut microbiota may result in the development of other diseases such as obesity, diabetes and hypertension (16, 17).

Diarrhea is deemed as the major factor impeding the development of livestock industry because of it could cause reduced growth performance and a large number of deaths of farmed animals (18, 19). Increasing surveys demonstrated that diarrhea occurs in almost all animals, causing huge economic losses every year (20, 21). Diarrhea could cause the body to lose a great of water and nutrients, thus resulting in energy imbalances, weakness, starvation or even death (22, 23). Moreover, it may also cause inappetence, lassitude and weight loss, seriously affecting animal health and growth performance (24). Given the adverse effect of diarrhea on the livestock industry, investigating its treatment and etiology is of great significance. Research showed that gut microbial community is closely related to the development of diarrhea (14). For instance, early studies indicated that the compositions and structures of gut bacterial and fungal communities changed significantly in many diarrheal mammals (25, 26). Additionally, fecal microbiota transplantation was shown to alleviate diarrhea in some exploratory experiments, suggesting important roles of gut microbiota in diarrheal prevention and control (8).

Recently, metagenomics has developed into an effective tool for analyzing the gut microbiota (27, 28). By deep sequencing of intestinal contents or fecal samples, we can reveal the complex composition of the gut microbiota and explore gut microbial changes during disease, which contribute to understanding the pathological mechanism of disease and the role of gut microbiota (29, 30). Meanwhile, it also beneficial to diagnose and treat diseases from the microbiological perspective and decrease animal mortality and economic losses (31, 32). Horses (*Equus caballus*) are closely related to human life and development. In the past, the main uses of horses included meat, dairy, agricultural production, transportation, and the military. With the development of society, horses are mainly used for sports entertainment at this stage. However, horses are prone to diarrheal diseases due to stress response, excessive exercise and other factors. Currently, the characteristics of gut microbiota in many diarrheic animals such as giraffe, sheep and pigs have been detected by amplicon sequencing and revealed the changes in gut microbiota (14, 33). However, knowledge regarding diarrhea influence on gut microbiota in horses remains scarce. Here, we investigated the alterations of gut fungal community in diarrheic horses.

## Materials and methods

### Sample acquisition

In this research, 16 fecal sample collected from eight healthy and eight diarrheic horses were applied for amplicon sequencing. These horses were raised at the Wuhan Business University (Wuhan, China) and have similar age (~2 years old) and breeding conditions. Prior to sample acquisition, the diarrheic horses were assessed and diagnosed by professional veterinarian. To collect clean samples, the sterile swabs were used for swabbing rectum in a rotating fashion. The collected fecal sample were immediately sub-sampled from the intermediate region to maximally decrease pollution and then snap-frozen utilizing liquid nitrogen and stored at −80°C for further study.

### DNA extraction and illumine MiSeq sequencing

Fecal sample collected from different groups were thawed and then subjected to bacterial genome DNA extraction using QIAamp DNA Mini Kit (QIAGEN, Hilden, Germany) based on the manufacturer's instructions. The gDNA were subjected to quality evaluation *via* using 0.8% (w/v) agarose gel electrophoresis, while its concentration was quantified by using UV-Vis spectrophotometer (NanoDrop 2000, United States). Subsequently, we amplified the V3/V4 regions of 16S rRNA using the primers (338F: ACTCCTACGGGAGGCAGCA and 806R: GGACTACHVGGGTWTCTAAT) synthesized from conserved regions. PCR amplification procedure was determined based on previous reports (34). The amplified products were conducted quality inspection, target fragment recovery, fluorescent quantitation and purification. The PacBio platform (Biomarker Technologies, China) was applied to construct sequencing libraries and qualified libraries were paired-end sequenced on MiSeq sequencing machine according to the standard protocols. The raw data generated by amplicon sequencing were filtered and identified to eliminate short sequences, mismatched primers and chimera. After quality assessment, the qualified sequences were clustered and OTUs partitioned based on 97% similarity. Moreover, we also calculated multiple alpha diversity indices and generated PCoA plots to further dissect changes in gut microbial abundance, diversity and principal components. Metastats analysis and LEfSe were used to distinguish differential taxa. *P*-values (means ± SD) < 0.05 were considered statistically significant.

## Results

### Data acquisition and analysis

**Figure 1:**
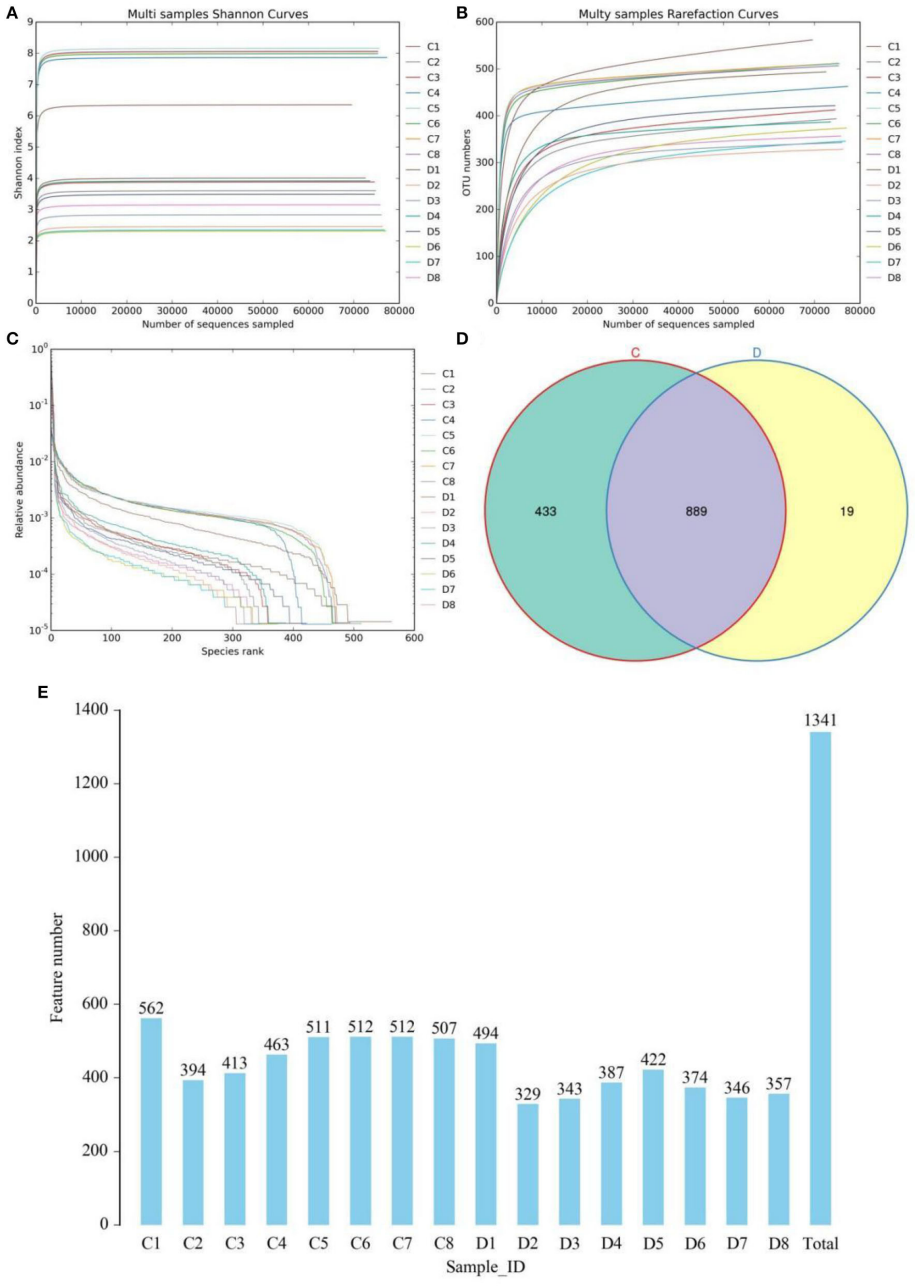
Gut fungal feasibility analysis and OTUs distribution. Rarefaction curves **(A,B)** and rank abundance curve **(C)** were used to evaluate the sequencing depth and evenness. **(D)** Venn diagrams for core and unique OTUs in the healthy and diarrheic groups. Histogram showing the number of OTUs in each sample. **(E)** Histogram showing the number of OTUs in each sample. C and D represent healthy and diarrheic groups, respectively.

In this study, we amplified 16 fecal samples from healthy and diarrheic horses to assess changes in the gut fungal community during diarrhea. Results of amplicon sequencing indicated that 127,8594 (C = 639,936, D = 638,658) raw sequences were totally generated, with an average of 79,912 (varying from 79,473 to 80,221) sequences per sample (Table 1). After quality evaluation, a total of 1252,897 (C = 626,347, D = 626,550) qualified sequences were collected, with a qualification rate of over 60%. The rarefaction curve and species rank curve in each sample were wide and decreased slowly, showing the satisfactory sequencing evenness and richness (Figures 1A–C). According to 97% nucleotide-sequence similarity, the qualified sequences were clustered into 1341 OTUs, ranging from 329 to 562 OTUs per sample (Figures 1D,E). Among identified OTUs, 889 OTUs are common in both groups, accounting for approximately 66.29% of the total OTUs. Moreover, there are 433 and 19 unique OTUs in the healthy and diarrheic horses.

**Table 1:** Gut fungal sequence data of the samples.

**Sample**	**Raw reads**	**Clean reads**	**Effective reads**	**AvgLen (bp)**	**GC** **(%)**	**Q20** **(%)**	**Q30** **(%)**	**Effective (%)**
C1	80175	79788	76634	290	47.5	99.6	98.1	95.6
C2	79705	79297	78271	286	29.9	99.8	98.6	98.2
C3	79961	79540	78572	284	30.8	99.8	98.7	98.3
C4	80220	79801	78919	249	48.4	99.9	99.2	98.4
C5	79848	79414	78392	244	48.3	99.9	99.2	98.2
C6	79956	79514	78475	249	48.4	99.9	99.1	98.2
C7	80221	79823	78541	249	48.3	99.9	99.2	97.9
C8	79850	79402	78543	251	48	99.9	99.1	98.4
D1	79827	79369	78020	286	33.2	99.8	98.5	97.7
D2	79937	79577	78621	292	31.2	99.8	98.6	98.4
D3	80128	79714	78775	290	31.2	99.8	98.6	98.3
D4	79757	79355	77485	285	34.2	99.8	98.6	97.2
D5	79473	79074	78208	289	31.8	99.8	98.5	98.4
D6	79973	79582	78755	293	30.3	99.8	98.5	98.5
D7	79809	79455	78625	294	30.6	99.8	98.5	98.5
D8	79754	79353	78061	292	29.8	99.8	98.5	97.9

### Diarrhea reduces the diversity of gut fungal community

**Figure 2:**
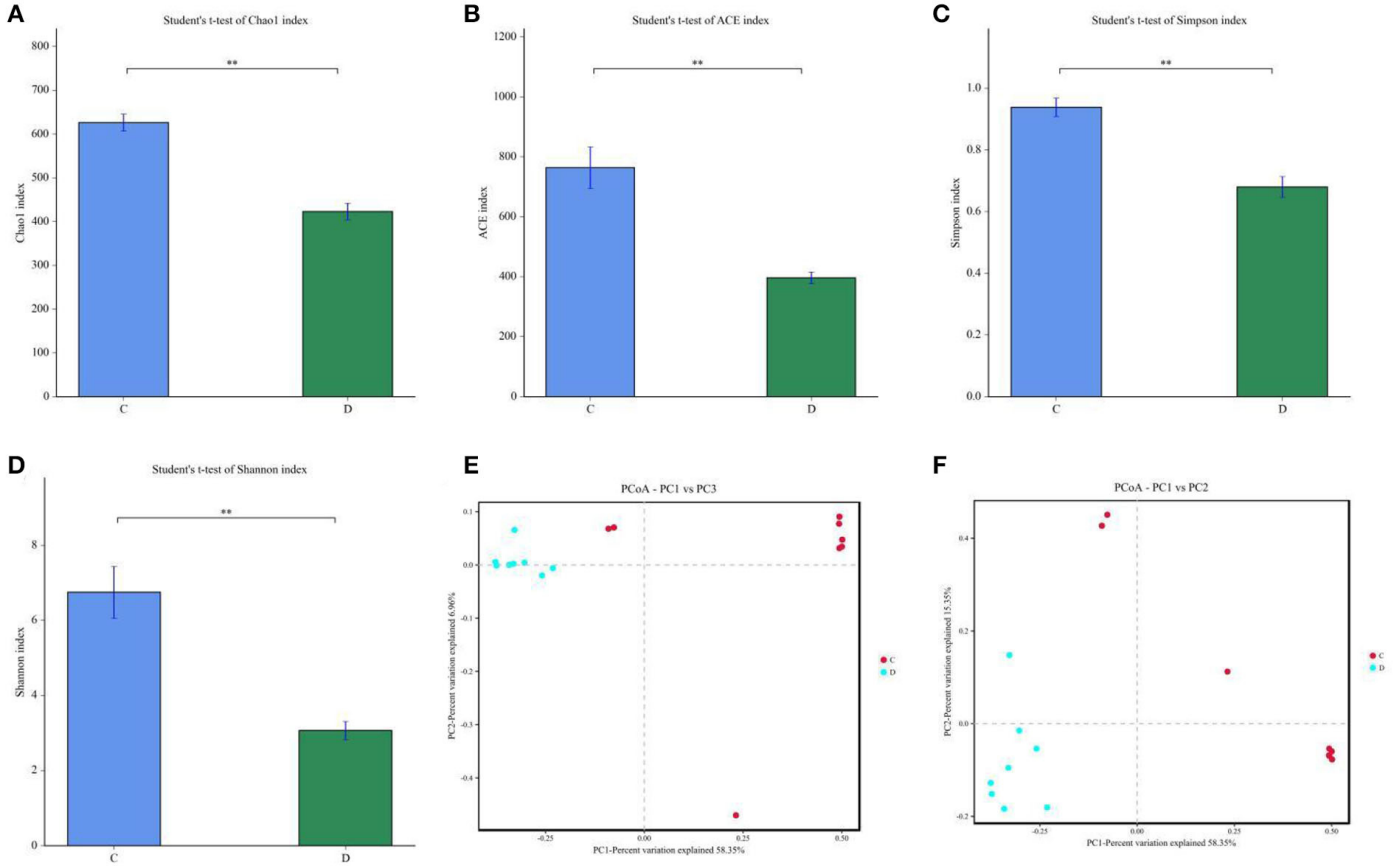
Effects of diarrhea on gut fungal diversity in horses. **(A–D)** represents the Chao1, ACE, Simpson and Shannon indices that can reflect the diversity of gut fungal community **(E,F)** Principal Coordinate Analysis (PCoA) of gut fungal community in healthy and diarrheic groups. C and D represent healthy and diarrheic groups, respectively. Data was presented as the mean ± SD. **P < 0.01.

To further investigate the influences of diarrhea in gut fungal community, we comparatively analyzed shifts in gut fungal diversity index between healthy and diarrheic horses. Good's coverage estimations in each sample ranged from 99.90 to 99.97%, covering nearly all fungal phenotypes. Statistical analysis of alpha diversity indicated that there were statistically distinct differences in the Chao1 (626.07 ± 54.84 vs. 422.88 ± 54.21, *P* < 0.01), ACE (763.48 ± 195.95 vs. 396.00 ± 54.65, *P* < 0.01), Simpson (0.93 ± 0.084 vs. 0.67 ± 0.094, *P* < 0.01) and Shannon (6.74 ± 1.94 vs. 3.06 ± 0.68, *P* < 0.01) indices between the control and diarrheic groups, indicating that diarrhea significantly reduced the gut fungal diversity and abundance (Figures 2A–D). Additionally, beta-diversity analysis showed that the dots in healthy and diarrheic group were separated, demonstrating that diarrhea dramatically changed the gut fungal main components (Figures 2E,F).

### Comparative analysis of the gut fungal composition between healthy and diarrheic groups

**Figure 3:**
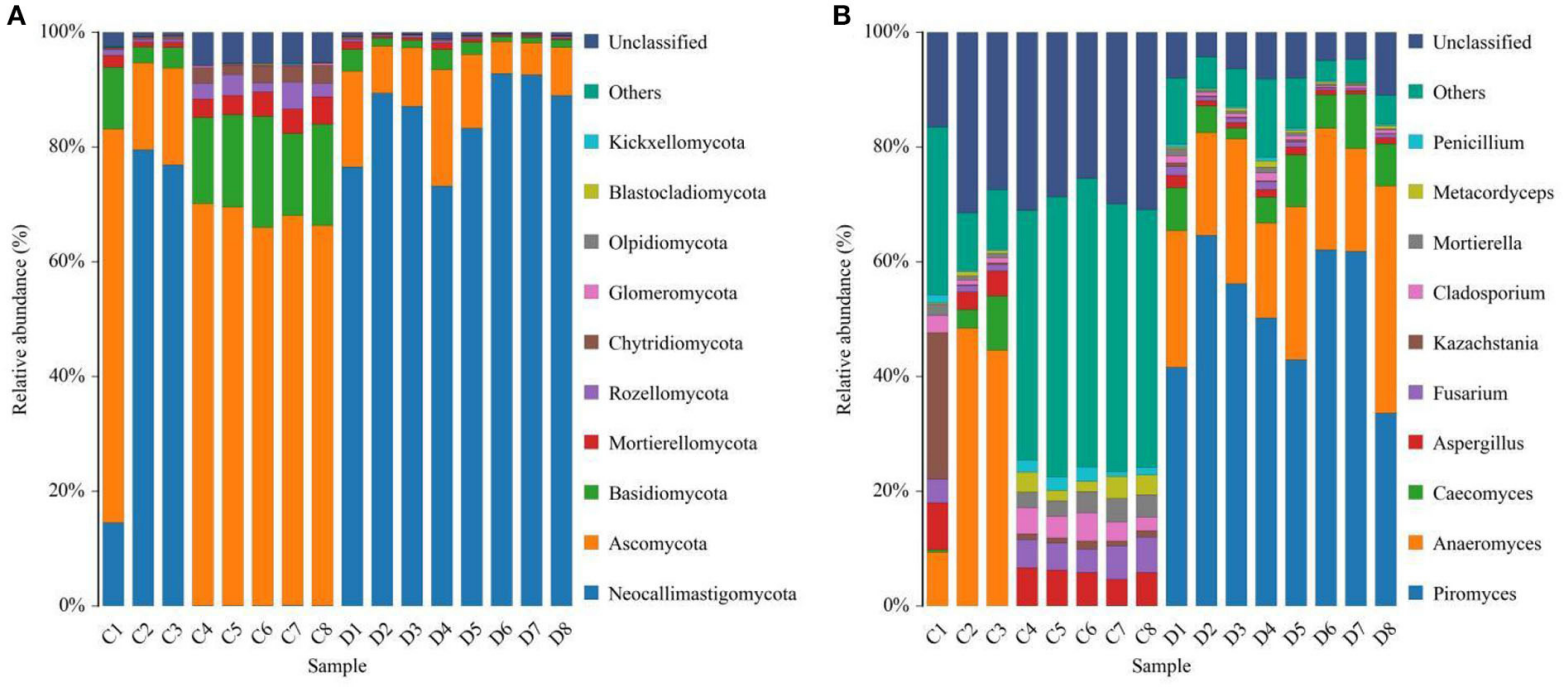
Effects of diarrhea on gut fungal composition in horses. **(A)** The composition of dominant fungi at the phylum level. **(B)** The composition of dominant fungi at the genus level. C and D represent healthy and diarrheic groups, respectively.

**Figure 4:**
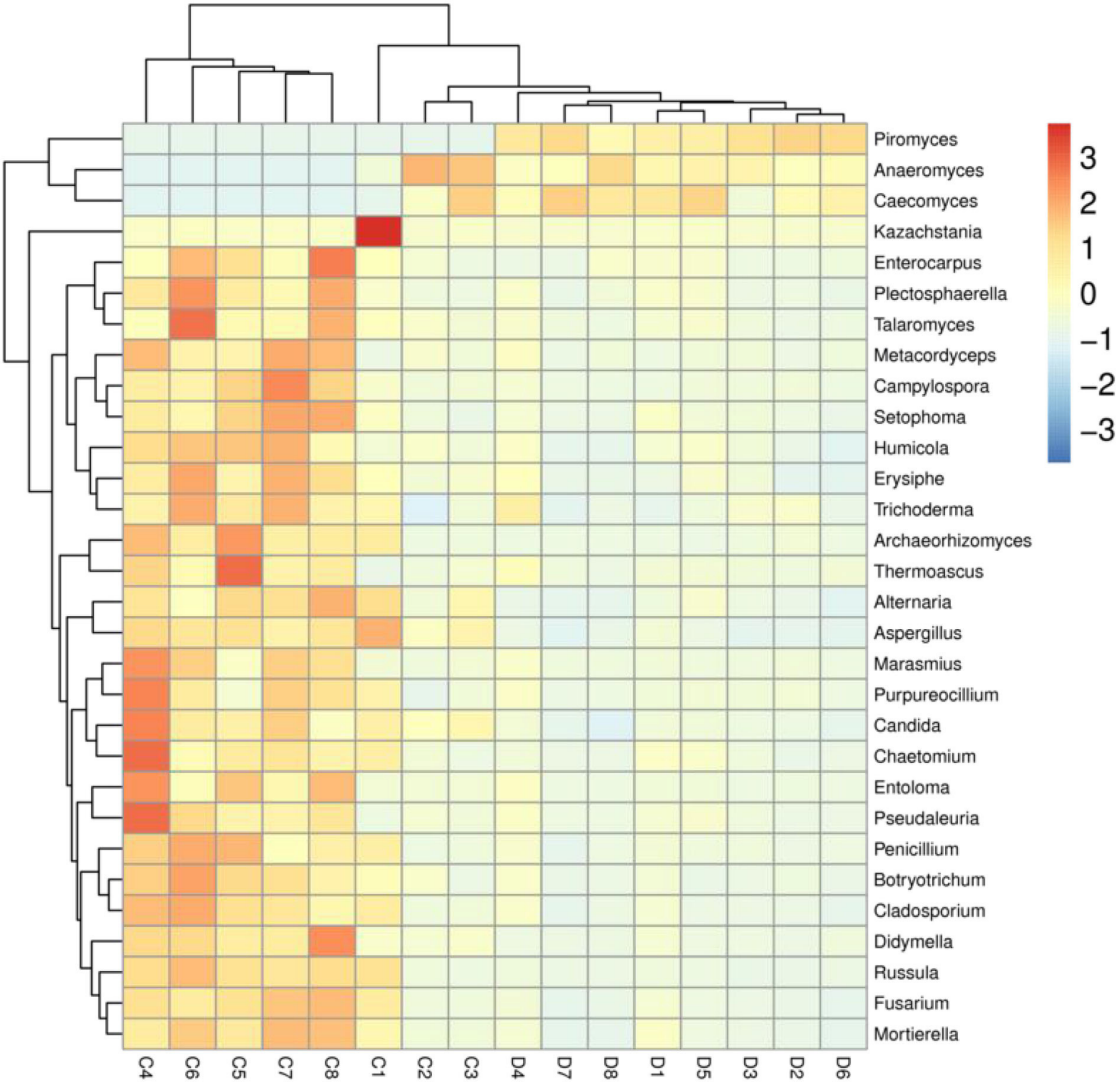
Heatmap of the most abundant fungal genera in both groups. The values of color in the heat map indicate the normalized relative richness of species. C and D represent healthy and diarrheic groups, respectively.

We also visualized the composition and abundance of gut fungal community at different taxonomical levels through species distribution histograms and observed considerable variability. There were nine phyla and 119 genera detected in 16 samples, ranging from 5 to 7 phyla per sample. The phyla *Neocallimastigomycota* (21.30%, 85.59%), *Ascomycota* (55.01%, 10.87%), and *Basidiomycota* (12.49%, 1.89%) were abundantly present in the healthy and diarrheic groups, accounting for over 80% of total taxonomic groups recognized (Figure 3A). Other phyla such as *Chytridiomycota* (1.83%, 0.22%), *Glomeromycota* (0.16%, 0.018%), *Olpidiomycota* (0.060%, 0.0051%), *Blastocladiomycota* (0.031%, 0.00%), and *Kickxellomycota* (0.023%, 0.00%) in healthy and diarrheic groups were recognized in low abundances. Among identified genera, *Anaeromyces* (12.74%), *Aspergillus* (5.56%), *Fusarium* (4.00%), and *Kazachstania* (3.68%) were the four predominant fungal genus in the control groups, accounting for more than 20.00% of the total composition (Figure 3B). Furthermore, the dominant fungal genus observed in gut fungal community in the diarrheic group were *Piromyces* (51.73%), *Anaeromyces* (23.61%), *Caecomyces* (6.29%), and *Aspergillus* (1.14%), accounting for over 80.00% of the total composition. Additionally, gut fungal distribution and variability between both groups could also be observed by the visualized clustering heatmap (Figure 4).

**Figure 5:**
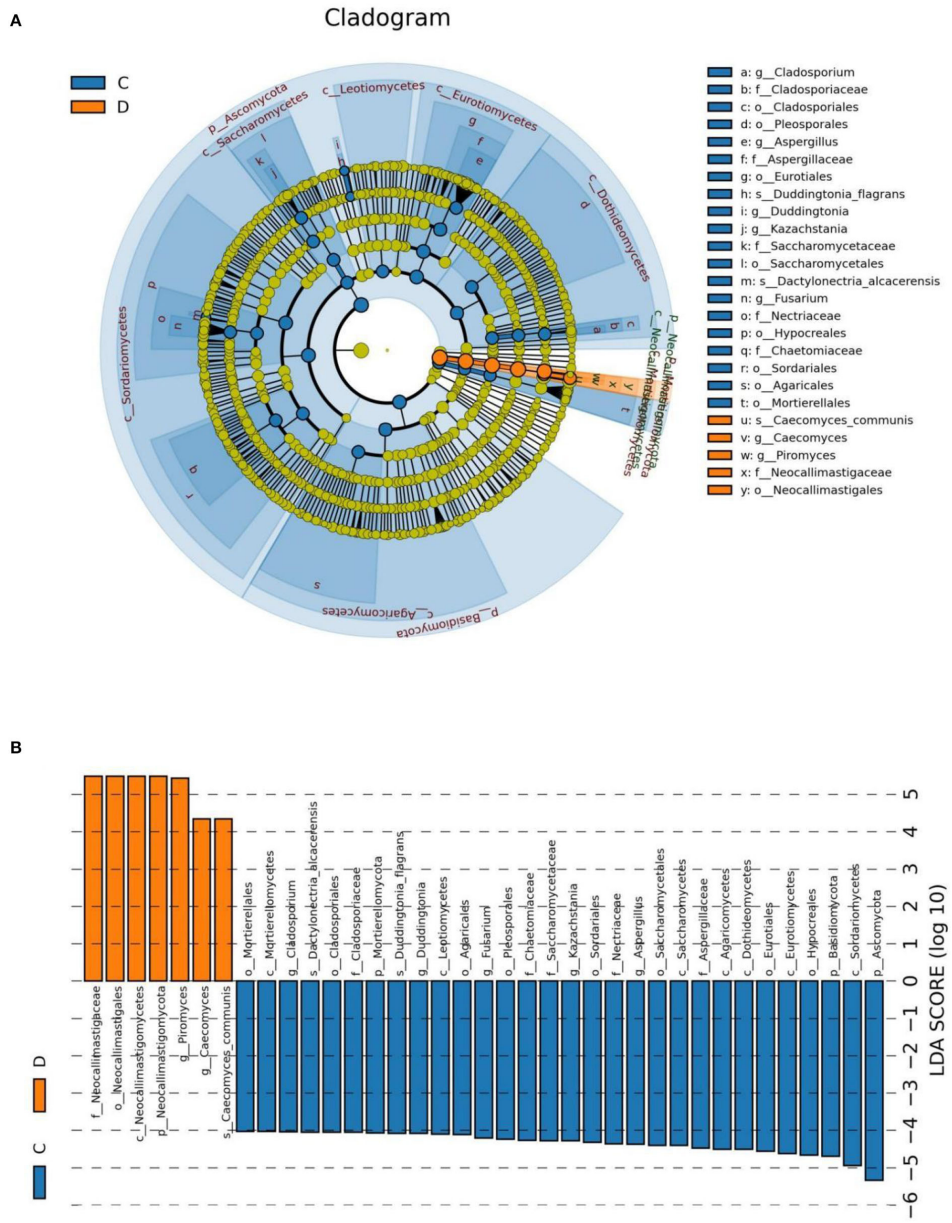
The identification of differential fungi associated with diarrhea. **(A)** Cladogram indicating the phylogenetic distribution of microbiota correlated with the healthy or diarrheic horses. **(B)** The differences in abundance between the healthy and diarrheic horses. C and D represent healthy and diarrheic groups, respectively.

At the phylum level, *Blastocladiomycota, Kickxellomycota, Rozellomycota, Ascomycota, Basidiomycota, Chytridiomycota, Mortierellomycota, Neocallimastigomycota, Glomeromycota*, and *Olpidiomycota* in the healthy group were significantly preponderant than diarrheic group (Table 2). Moreover, we also observed that 175 fungal genera were significantly different between the control and diarrheic groups (Supplementary Table 1). Among these differential taxa, the relative abundances of 171 fungal genera (*Achroiostachys, Acremoniopsis, Alatospora, Alternaria, Apodus, Arthrocladium, Aschersonia, Ascobolus, Aspergillus, Bartalinia, Berkleasmium, Brunneomyces, Butlerelfia, Candida, Coprinellus, Coryne, Craterellus, Cystodermella, Deconica, Didymella, Duddingtonia, Edenia, Entrophospora, Eremothecium, Erysiphe, Gamsia, Geminibasidium, Golovinomyces, Grammothele, Graphilbum, Gymnopilus, Hanseniaspora, Hypholoma, Kalmusia, Keissleriella, Leohumicola, Leucoagaricus, Leucosphaerina, Limnoperdon, Linderina, Microdochium, Microglossum, Minutisphaera, Monocillium, Myxospora, Panaeolus, Paraconiothyrium, Paracremonium, Phomatospora, Pleuroascus, Polyscytalum, Porodiplodia, Psathyrella, Pseudocoleophoma, Psilocybe, Pyrenochaeta, Ramichloridium, Ramularia, Rigidoporus, Roussoella, Scedosporium, Schizophyllum, Sclerostagonospora, Sebacina, Simplicillium, Spizellomyces, Sporormiella, Stellatospora, Strelitziana, Taifanglania, Talaromyces, Torula, Toxicocladosporium, Trichomerium, Trichometasphaeria, Trichomonascus, Trichophyton, Ustilaginoidea, Uwebraunia, Vishniacozyma, Wickerhamomyces, Wilcoxina, Xanthothecium, Archaeorhizomyces, Botryotrichum, Campylospora, Chaetomium, Fusarium, Kazachstania, Meyerozyma, Mortierella, Paecilomyces, Pichia, Russula, Saitozyma, Trichosporon, Cladosporium, Coniochaeta, Oidiodendron, Penicillium, Wallemia, Chaetomidium, Cladorrhinum, Hannaella, Humicola, Schizothecium, Trichocladium, Cephalotrichum, Entoloma, Ophiostoma, Setophoma, Acremonium, Enterocarpus, Sampaiozyma, Apiotrichum, Articulospora, Debaryomyces, Gibellulopsis, Lasiobolidium, Lecanicillium, Marquandomyces, Solicoccozyma, Staphylotrichum, Plectosphaerella, Pseudogymnoascus, Condenascus, Marasmius, Cladophialophora, Olpidium, Epicoccum, Pseudaleuria, Thelephora, Dactylonectria, Metacordyceps, Chrysosporium, Inosperma, Podospora, Rhodotorula, Pyrenochaetopsis, Monascus, Neomicrosphaeropsis, Tetracladium, Trechispora, Purpureocillium, Tausonia, Cercophora, Cercospora, Fusariella, Pseudeurotium, Thanatephorus, Preussia, Teunomyces, Exophiala, Amphinema, Cortinarius, Filobasidium, Zygosaccharomyces, Arxiella, Trichoderma, Thermoascus, Paraphaeosphaeria, Myriococcum, Triangularia, Cylindrobasidium, Knufia, Symmetrospora, Fusicolla, Byssochlamys, Gliocladiopsis, Stagonosporopsis*, and *Trametes*) dramatically decreased, whereas the relative richness of four fungal genera (*Claviceps, Piromyces, Geosmithia*, and *Caecomyces*) significantly increased during diarrhea. Notably, 74 genera even cannot be detected in the gut fungal community of diarrheic horses. Considering this discriminant analysis cannot found all the taxon, LEfSe combined with LDA scores were used to recognize the specific fungi associated with diarrhea (Figure 5).

**Table 2:** Comparative analysis of differential fungal phyla between control and diarrheic groups.

**Taxa**	**C (%)**	**D (%)**	**P**
*Blastocladiomycota*	0.0313 ± 0.0213	0 ± 0	0
*Kickxellomycota*	0.0229 ± 0.0229	0 ± 0	0
*Rozellomycota*	2.13 ± 0.54	0.287 ± 0.0373	0
*Ascomycota*	55± 8.53	11 ± 1.87	0
*Basidiomycota*	12.5 ± 2.2	1.92 ± 0.409	0
*Chytridiomycota*	1.82 ± 0.437	0.226 ± 0.0446	0
*Mortierellomycota*	2.95 ± 0.543	0.578 ± 0.146	0
*Neocallimastigomycota*	21.4 ± 12.5	85.5 ± 2.57	0
*Glomeromycota*	0.165 ± 0.0509	0.0178 ± 0.00765	0.01
*Olpidiomycota*	0.0605 ± 0.0237	0.0052 ± 0.00218	0.01

C and D represent healthy and diarrheic groups, respectively.

### Correlation network analysis

**Figure 6:**
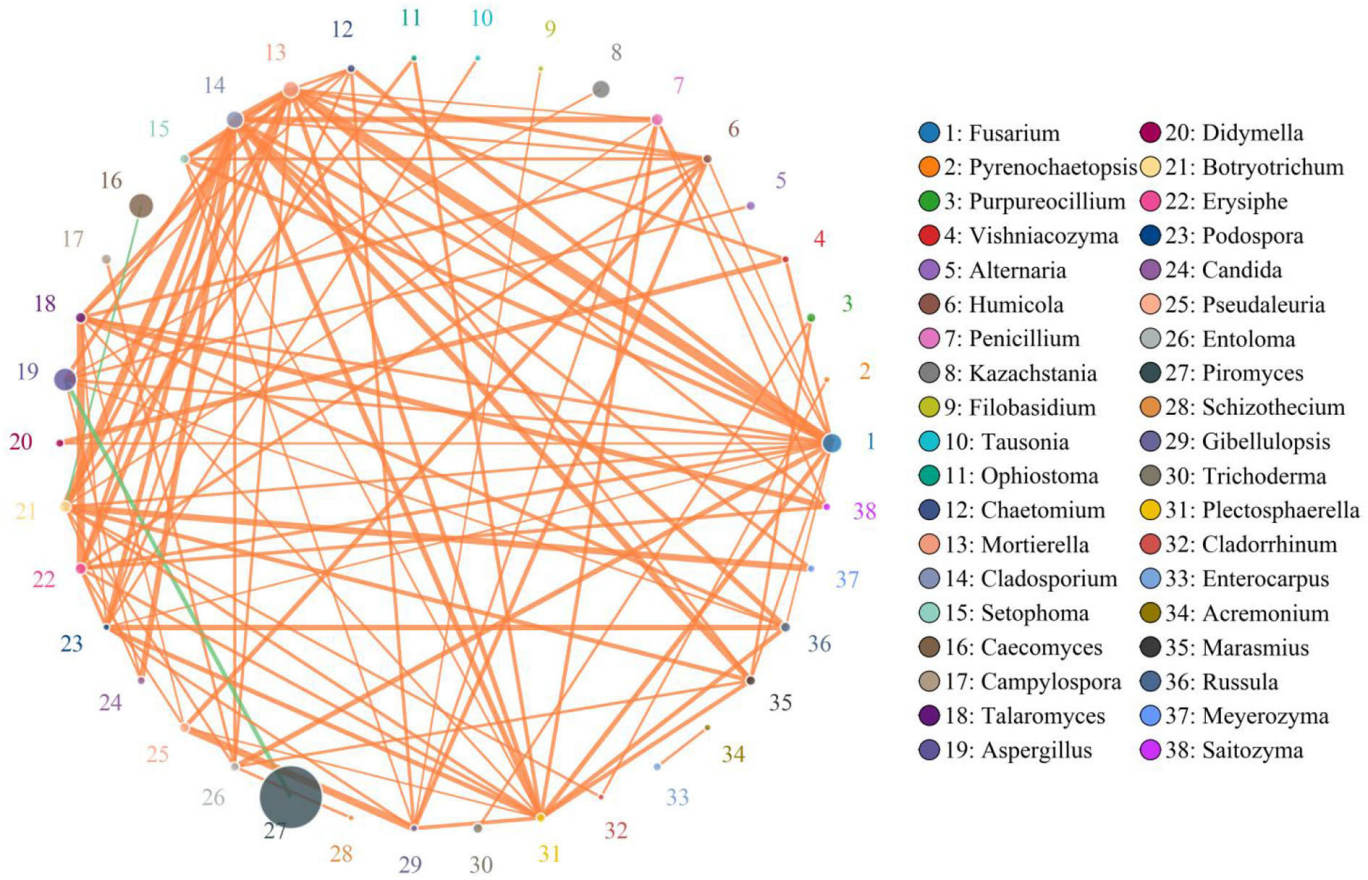
Correlation network analysis of gut fungal diversity. The correlation between fungi is represented by lines of different colors. The orange lines represent the positive correlation, whereas the green lines represent negative correlation. Detailed data were shown in the Supplementary Table 2.

*Erysiphe* was positively associated with *Campylospora* (0.8706), *Russula* (0.8735), *Pseudaleuria* (0.8824), *Trichoderma* (0.7882), *Setophoma* (0.8471), *Marasmius* (0.8676), *Purpureocillium* (0.8324), *Alternaria* (0.8176), *Humicola* (0.8971), *Entoloma* (0.8500), *Plectosphaerella* (0.8941), *Enterocarpus* (0.6706), *Thermoascus* (0.6529), *Chaetomium* (0.8529), *Didymella* (0.8000), *Candida* (0.8735), *Archaeorhizomyces* (0.6412), *Saitozyma* (0.8971), *Meyerozyma* (0.7765), *Vishniacozyma* (0.8176), *Thelephora* (0.8029), *Rhodotorula* (0.7059), *Trichocladium* (0.6559), *Gibellulopsis* (0.8706), *Sebacina* (0.7640), *Debaryomyces* (0.5618), *Paecilomyces* (0.7206), *Acremonium* (0.7559), *Tetracladium* (0.6412), *Lecanicillium* (0.6785), *Pseudeurotium* (0.6500), *Ophiostoma* (0.7794), *Condenascus* (0.7176), *Podospora* (0.8971), *Solicoccozyma* (0.7108), *Preussia* (0.6176), *Schizophyllum* (0.8529), *Fusariella* (0.6882), *Monascus* (0.8647), *Trichosporon* (0.7647), *Cephalotrichum* (0.8176), *Tausonia* (0.6902), *Pyrenochaetopsis* (0.5765), *Cladorrhinum* (0.6941), *Trechispora* (0.6941), *Staphylotrichum* (0.6735), *Coniochaeta* (0.7029), *Oidiodendron* (0.7618), *Cercospora* (0.5471), *Filobasidium* (0.7882), *Zygosaccharomyces* (0.6896), and *Schizothecium* (0.7403) (Figure 6). *Podospora* was positively related to *Solicoccozyma* (7285), *Schizophyllum* (0.7588), *Fusariella* (0.8618), *Monascus* (0.7324), *Trichosporon* (0.7588), *Cephalotrichum* (0.7118), *Tausonia* (0.8859), *Pyrenochaetopsis* (0.7176), *Cladorrhinum* (0.7500), *Trechispora* (0.6882), *Staphylotrichum* (0.5853), *Coniochaeta* (0.6706), *Oidiodendron* (0.6588), *Cercospora* (0.5941), *Filobasidium* (0.8324), *Zygosaccharomyces* (0.8031) and *Schizothecium* (0.6652). *Fusarium* was positively associated with *Kazachstania* (0.8353), *Cladosporium* (0.9147) *Mortierella* (0.9794), *Metacordyceps* (0.6971), *Penicillium* (0.8824), *Botryotrichum* (0.8882), *Erysiphe* (0.8971), *Talaromyces* (0.8941), *Campylospora* (0.8382), *Russula* (0.8765), *Pseudaleuria* (0.8618), *Trichoderma* (0.7059), *Setophoma* (0.9118), *Marasmius* (0.8824), *Purpureocillium* (0.8059), *Alternaria* (0.8647), *Humicola* (0.8794), *Entoloma* (0.9176), *Plectosphaerella* (0.9147), *Enterocarpus* (0.7294), *Thermoascus* (0.7029), *Chaetomium* (0.9176), *Didymella* (0.8794), *Cadida* (0.8529), *Archaeorhizomyces* (0.7147), *Saitozyma* (0.8618), *Meyerozyma* (0.8441), *Vishniacozyma* (0.8941), *Thelephora* (0.7765), *Rhodotorula* (0.75), *Trichocladium* (0.7324), *Gibellulopsis* (0.8559), *Sebacina* (0.823), *Debaryomyces* (0.5176), *Paecilomyces* (0.7618), *Acremonium* (0.7941), *Tetracladium* (0.7647), *Lecanicillium* (0.4993), *Pseudeurotium* (0.5412), *Ophiostoma* (0.8294), *Condenascus* (0.7029), *Podospora* (0.8765), *Solicoccozyma* (0.7403) and *Preussia* (0.5529). *Schizophyllum* (0.7559), *Fusariella* (0.6912), *Monascus* (0.7294), *Trichosporon* (0.8324), *Cephalotrichum* (0.7676), *Tausonia* (0.727), *Pyrenochaetopsis* (0.8029), *Cladorrhinum* (0.8324), *Trechispora* (0.8088), *Staphylotrichum* (0.6059), *Epicoccum* (0.5882), *Coniochaeta* (0.7382), *Oidiodendron* (0.7353), *Filobasidium* (0.7235), *Zygosaccharomyces* (0.6866), *Leptobacillium* (0.6206), *Schizothecium* (0.8109), *Knufia* (0.5158) and *Thanatephorus* (0.6342).

## Discussion

Numerous studies have indicated that gut microbiota played key roles in resisting the invasion of pathogenic bacteria and regulating the intestinal homeostasis, whereas gut microbial dysbiosis may cause many gastrointestinal disease or even systemic effects (35–37). Therefore, investigating thoroughly the changes of gut microbiota in different health status is of great significance for the disease prevention and treatment. Meanwhile, it also contributes to understanding the pathogenesis and developing novel methods for diagnosing the disease (38, 39). Diarrhea is a common gastrointestinal disease present in various animals, which results in a enormous threat to economic development, livestock production and animal welfare (20). Notably, the prevention and control of diarrhea-related diseases is difficult due to many factors including pathogenic bacteria, oxidative stress, environmental change and intoxication (40, 41). Diarrhea is often accompanied by intestinal damage, suggesting that the gut microbiota is inevitably affected by diarrhea (42, 43). Presently, many studies have been performed to explore gut microbial changes in sheep, yaks, dogs and chickens during diarrhea, but little is known about the characteristics of gut microbiota in diarrheic horse. In this study, we first systematically explored the shifts of gut fungal community in diarrheic horse.

Gut microbiota is a complex and dynamically changing system that is susceptible to a many factors including elevation, temperature, age, diet and species (44–46). However, this normal fluctuation of gut microbiota does not affect the execution of complex intestinal functions (47, 48). However, strong stimulus such as various gastrointestinal diseases, antibiotics, heavy metal and microplastics could cause significant variation in gut microbial diversity and composition, causing gut microbial dysbiosis (49–51). Increasing investigations showed that the occurrence of diarrhea is often accompanied by significant changes in the gut microbiota. For instance, Li et al. showed that diarrhea can cause dramatic changes in gut microbial composition as well as obvious reduction in gut microbial diversity (14). In addition, Hong et al. the gut fungal diversity of patients with diarrhea-predominant irritable bowel syndrome was dramatically different from that of healthy population (52). Therefore, gut fungal community of diarrheal horses may also undergo significant changes. Consistent with previous investigations, this research demonstrated an significantly reduced alpha-diversity indices in the gut fungal community of diarrheal horses, indicating gut microbial dysbiosis. As an important component of the gut microbiota, gut fungal community also plays key roles in intestinal homeostasis and function (53). Early investigations indicated that the execution of intestinal function depended on normal gut microbiota, thus disordered gut microbiota inevitably affected the intestinal functions including energy utilization, nutrient absorption and metabolism (46, 53, 54). Moreover, disrupted gut microbial homeostasis also affects intestinal barrier function and mucosal immunity, which may increase disease susceptibility (7, 55, 56). Consequently, diarrheic horses may be infected with other diseases during gut microbial imbalance. PCoA was also conducted to evaluate the shifts in gut microbial main components to further explore the effects of diarrhea on the equine gut microbiota. We found that the individuals of the control group were clustered together but separated from the diarrheic group, suggesting obvious alternations in the gut microbial main components. The present research showed that despite of shared environment and diets, the diarrheic horses displayed obvious changes in the gut fungal community. Thus, we suspected that diarrhea was the primary driving force for shifts in gut fungal community of horses.

Our results revelaed that *Basidiomycota* and *Ascomycota* were detected to be abundantly presented in healthy and diarrheic horses. Notably, these fungal phyla were also the most abundant fungal phyla in other species such as giraffe, yak and sheep, showing their key roles in intestinal ecology and function (14). We further explored the changes of gut fungal abundance of diarrheic horses. The relationship between diarrhea and gut microbial community could be intuitively reflected by some specific bacteria and fungi. This study indicated obvious declines in the relative abundances of 10 fungal phyla during diarrhea in horses. Moreover, although the species of the dominant phyla did not alter, the relative abundances of *Basidiomycota* and *Ascomycota* were significantly decreased, suggesting gut fungal dysbiosis. Importantly, we also observed high variations in some fungal genera during diarrhea and these changed fungal genera may play important roles in intestinal homeostasis and functions. Among the altered fungi, more than 97% of the fungal genera were significantly decreased in abundances and 74 fungal genera even cannot be detected in the gut fungal community of diarrheic horses, suggesting that these fungal genera cannot adapt to the current intestinal environment. We suspected that intestinal environment was disrupted during diarrhea, which in turn limited the survival of these fungi.

Increasing evidence indicated that the disruption of gut microbial homeostasis was a pathological mediator of various gastrointestinal diseases (57–59). The interaction between gut microbes, including bacteria and fungi, is an important way to maintain intestinal homeostasis (37, 60, 61). Typically, these interreaction include synergy, antagonism and commensalism (61). Therefore, the shifts of some microorganisms could affect the other microbial functions, thereby further exaggerating the overall influence of gut microbiota on the host health and causing gut microbial dysbiosis (33, 62). In this study, we also observed significant correlations between some fungi by correlation network analysis, indicating that these altered fungi could affect other gut fungal functions. This research indicated that diarrhea not only directly destroyed the gut fungal composition and structure but also impaired the other fungal functions *via* interactions, which may further result in gut fungal dysbiosis in diarrheal horses.

In conclusion, this study compared and analyzed the differences in the gut fungal community of healthy and diarrheal horses. Results indicated that diarrhea dramatically altered gut fungal composition and structure, characterized by altered gut fungal diversity and composition. To our knowledge, this is the first study of gut fungal changes in diarrheal horses. This study filled a gap in the effect of diarrhea on the gut fungal community in horses and indicated that gut fungal dysbiosis may be one of the causes of diarrhea in horses. Meanwhile, the present results also provided a theoretical basis for the diagnosis and treatment of diarrhea from the gut microbial perspective. However, this study has some limitations including relatively small sample size and inability to control for potentially important variables such as individual variation and individual dietary habit.

## Data availability statement

The data presented in the study are deposited in the Sequence Read Archive (SRA) (NCBI, USA) repository, accession number PRJNA881595.

## Ethics statement

The animal study was reviewed and approved by the Ethics Committee of the Wuhan Business University.

## Author contributions

YLi and YLa conceived and designed the experiments. GY contributed sample collection and reagents preparation. ZZ analyzed the data. YLa wrote the manuscript. II revised the manuscript. All authors reviewed the manuscript.

## Funding

The study was supported by the Wuhan Business University project (No. 2019KY003).

## Conflict of interest

The authors declare that the research was conducted in the absence of any commercial or financial relationships that could be construed as a potential conflict of interest.

## Publisher's note

All claims expressed in this article are solely those of the authors and do not necessarily represent those of their affiliated organizations, or those of the publisher, the editors and the reviewers. Any product that may be evaluated in this article, or claim that may be made by its manufacturer, is not guaranteed or endorsed by the publisher.
