# Supplementary material for: Dynamic changes of gut fungal community in horse at different health states
Source: Front Vet Sci. 2022 Oct 28;9:1047412. doi: 10.3389/fvets.2022.1047412 (PMC9650549; doi:10.3389/fvets.2022.1047412)
Supplement: Supplementary file 1 [file Table_1.docx]

**Supplementary Table S1.** Comparative analysis of differential fungal genera between control and diarrheic groups.

| Taxa | C (%) | D (%) | P |
| --- | --- | --- | --- |
| *Achroiostachys*  *Acremoniopsis*  *Alatospora*  *Alternaria*  *Apodus*  *Arthrocladium*  *Aschersonia*  *Ascobolus*  *Aspergillus*  *Bartalinia*  *Berkleasmium*  *Brunneomyces*  *Butlerelfia*  *Candida*  *Claviceps*  *Coprinellus*  *Coryne*  *Craterellus*  *Cystodermella*  *Deconica*  *Didymella*  *Duddingtonia*  *Edenia*  *Entrophospora*  *Eremothecium*  *Erysiphe*  *Gamsia*  *Geminibasidium*  *Golovinomyces*  *Grammothele*  *Graphilbum*  *Gymnopilus*  *Hanseniaspora*  *Hypholoma*  *Kalmusia*  *Keissleriella*  *Leohumicola*  *Leucoagaricus*  *Leucosphaerina*  *Limnoperdon*  *Linderina*  *Microdochium*  *Microglossum*  *Minutisphaera*  *Monocillium*  *Myxospora*  *Panaeolus*  *Paraconiothyrium*  *Paracremonium*  *Phomatospora*  *Piromyces*  *Pleuroascus*  *Polyscytalum*  *Porodiplodia*  *Psathyrella*  *Pseudocoleophoma*  *Psilocybe*  *Pyrenochaeta*  *Ramichloridium*  *Ramularia*  *Rigidoporus*  *Roussoella*  *Scedosporium*  *Schizophyllum*  *Sclerostagonospora*  *Sebacina*  *Simplicillium*  *Spizellomyces*  *Sporormiella*  *Stellatospora*  *Strelitziana*  *Taifanglania*  *Talaromyces*  *Torula*  *Toxicocladosporium*  *Trichomerium*  *Trichometasphaeria*  *Trichomonascus*  *Trichophyton*  *Ustilaginoidea*  *Uwebraunia*  *Vishniacozyma*  *Wickerhamomyces*  *Wilcoxina*  *Xanthothecium*  *Archaeorhizomyces*  *Botryotrichum*  *Campylospora*  *Chaetomium*  *Fusarium*  *Kazachstania*  *Meyerozyma*  *Mortierella*  *Paecilomyces*  *Pichia*  *Russula*  *Saitozyma*  *Trichosporon*  *Cladosporium*  *Coniochaeta*  *Oidiodendron*  *Penicillium*  *Wallemia*  *Chaetomidium*  *Cladorrhinum*  *Hannaella*  *Humicola*  *Schizothecium*  *Trichocladium*  *Cephalotrichum*  *Entoloma*  *Ophiostoma*  *Setophoma*  *Acremonium*  *Enterocarpus*  *Sampaiozyma*  *Apiotrichum*  *Articulospora*  *Debaryomyces*  *Geosmithia*  *Gibellulopsis*  *Lasiobolidium*  *Lecanicillium*  *Marquandomyces*  *Solicoccozyma*  *Staphylotrichum*  *Plectosphaerella*  *Pseudogymnoascus*  *Caecomyces*  *Condenascus*  *Marasmius*  *Cladophialophora*  *Olpidium*  *Epicoccum*  *Pseudaleuria*  *Thelephora*  *Dactylonectria*  *Metacordyceps*  *Chrysosporium*  *Inosperma*  *Podospora*  *Rhodotorula*  *Pyrenochaetopsis*  *Monascus*  *Neomicrosphaeropsis*  *Tetracladium*  *Trechispora*  *Purpureocillium*  *Tausonia*  *Cercophora*  *Cercospora*  *Fusariella*  *Pseudeurotium*  *Thanatephorus*  *Preussia*  *Teunomyces*  *Exophiala*  *Amphinema*  *Cortinarius*  *Filobasidium*  *Zygosaccharomyces*  *Arxiella*  *Trichoderma*  *Thermoascus*  *Paraphaeosphaeria*  *Myriococcum*  *Triangularia*  *Cylindrobasidium*  *Knufia*  *Symmetrospora*  *Fusicolla*  *Byssochlamys*  *Gliocladiopsis*  *Stagonosporopsis*  *Trametes* | 0.031±0.030  0.029±0.018  0.025±0.013  0.71±0.10  0.042±0.027  0.012±0.012  0.013±0.013  0.026±0.017  5.58±0.55  0.012±0.012  0.017±0.017  0.015±0.014  0.041±0.040  0.485±0.079  0±0  0.15±0.087  0.038±0.019  0.019±0.019  0.016±0.016  0.035±0.035  0.52±0.11  0.055±0.053  0.011±0.011  0.024±0.022  0.018±0.018  1.1±0.20  0.023±0.016  0.013±0.013  0.023±0.023  0.033±0.033  0.035±0.025  0.010±0.010  0.033±0.032  0.032±0.032  0.017±0.017  0.017±0.017  0.012±0.010  0.014±0.014  0.014±0.014  0.025±0.017  0.022±0.022  0.010±0.010  0.045±0.035  0.014±0.014  0.041±0.035  0.015±0.015  0.020±0.018  0.04±0.04  0.021±0.021  0.015±0.015  0.018±0.0023  0.012±0.012  0.012±0.012  0.015±0.015  0.020±0.016  0.010±0.010  0.033±0.022  0.018±0.018  0.034±0.034  0.017±0.017  0.015±0.015  0.018±0.018  0.029±0.019  0.24±0.077  0.019±0.018  0.34±0.087  0.011±0.011  0.027±0.024  0.022±0.017  0.010±0.010  0.022±0.022  0.088±0.056  0.99±0.27  0.014±0.014  0.046±0.031  0.061±0.045  0.034±0.027  0.012±0.012  0.021±0.021  0.029±0.029  0.059±0.029  0.35±0.058  0.016±0.016  0.021±0.021  0.012±0.0083  0.49±0.11  1.22±0.24  1±0.25  0.50±0.12  4±0.68  3.9±3.1  0.40±0.12  2.59±0.46  0.28±0.083  0.084±0.022  0.96±0.18  0.42±0.10  0.20±0.044  2.95±0.54  0.18±0.048  0.18±0.042  1.4±0.30  0.18±0.054  0.10±0.021  0.20±0.059  0.067±0.014  0.65±0.133  0.15±0.035  0.31±0.085  0.19±0.042  0.69±0.20  0.22±0.069  0.75±0.18  0.24±0.071  0.52±0.15  0.066±0.024  0.061±0.020  0.085±0.026  0.32±0.088  0.00018±0.00018  0.29±0.071  0.062±0.021  0.25±0.078  0.035±0.018  0.24±0.072  0.18±0.056  0.53±0.13  0.17±0.062  1.63±1.18  0.22±0.055  0.81±0.24  0.066±0.023  0.060±0.023  0.17±0.053  0.86±0.26  0.35±0.096  0.087±0.031  1.96±0.51  0.11±0.042  0.11±0.033  0.19±0.037  0.34±0.10  0.21±0.086  0.19±0.046  0.086±0.022  0.21±0.050  0.19±0.060  0.72±0.20  0.19±0.045  0.12±0.040  0.16±0.045  0.19±0.048  0.24±0.12  0.14±0.065  0.22±0.078  0.022±0.0069  0.051±0.021  0.10±0.051  0.11±0.035  0.14±0.031  0.16±0.047  0.036±0.015  0.70±0.14  0.48±0.14  0.042±0.021  0.052±0.024  0.016±0.0059  0.11±0.043  0.16±0.078  0.096±0.030  0.11±0.048  0.076±0.03  0.097±0.034  0.044±0.014  0.16±0.075 | 0±0  0±0  0±0  0.15±0.030  0±0  0±0  0±0  0±0  1.15±0.17  0±0  0±0  0±0  0±0  0.10±0.019  0.011±0.0091  0±0  0±0  0±0  0±0  0±0  0.077±0.013  0±0  0±0  0±0  0±0  0.30±0.071  0±0  0±0  0±0  0±0  0±0  0±0  0±0  0±0  0±0  0±0  0±0  0±0  0±0  0±0  0±0  0±0  0±0  0±0  0±0  0±0  0±0  0±0  0±0  0±0  51.6±4.02  0±0  0±0  0±0  0±0  0±0  0±0  0±0  0±0  0±0  0±0  0±0  0±0  0.02±0.0085  0±0  0.013±0.0056  0±0  0±0  0±0  0±0  0±0  0±0  0.22±0.046  0±0  0±0  0±0  0±0  0±0  0±0  0±0  0±0  0.083±0.012  0±0  0±0  0±0  0.049±0.01  0.26±0.054  0.14±0.023  0.12±0.028  0.84±0.14  0.2±0.072  0.046±0.018  0.54±0.14  0.063±0.015  0.0053±0.0019  0.076±0.018  0.10±0.015  0.042±0.010  0.72±0.13  0.028±0.0085  0.026±0.010  0.32±0.055  0.025±0.0087  0.025±0.0080  0.033±0.0087  0.011±0.0043  0.17±0.043  0.029±0.015  0.058±0.01  0.054±0.020  0.10±0.036  0.050±0.013  0.17±0.035  0.047±0.016  0.11±0.026  0.0015±0.0013  0.00066±0.00066  0.011±0.0038  0.030±0.0073  0.017±0.0071  0.077±0.013  0.00068±0.00051  0.030±0.017  0.00016±0.00016  0.027±0.0098  0.037±0.0077  0.16±0.030  0.012±0.0040  6.3±0.89  0.055±0.010  0.11±0.028  0.0057±0.0034  0.0052±0.0021  0.037±0.0096  0.16±0.050  0.070±0.021  0.013±0.0072  0.43±0.099  0.0042±0.0029  0.022±0.0092  0.077±0.024  0.072±0.019  0.032±0.011  0.062±0.012  0.018±0.0056  0.073±0.027  0.033±0.014  0.17±0.033  0.053±0.032  0.027±0.0084  0.042±0.012  0.066±0.017  0.040±0.0068  0.013±0.0046  0.047±0.016  0.0042±0.0027  0.0042±0.0041  0.010±0.0057  0.030±0.0096  0.061±0.014  0.035±0.021  0.0045±0.0030  0.30±0.071  0.15±0.03  0.00016±0.00016  0.0027±0.0018  0.0023±0.0011  0.030±0.013  0.010±0.0056  0.024±0.0096  0.012±0.0056  0.012±0.0062  0.019±0.0090  0.011±0.0041  0.021±0.0082 | 0.000999  0.000999  0.000999  0.000999  0.000999  0.000999  0.000999  0.000999  0.000999  0.000999  0.000999  0.000999  0.000999  0.000999  0.000999  0.000999  0.000999  0.000999  0.000999  0.000999  0.000999  0.000999  0.000999  0.000999  0.000999  0.000999  0.000999  0.000999  0.000999  0.000999  0.000999  0.000999  0.000999  0.000999  0.000999  0.000999  0.000999  0.000999  0.000999  0.000999  0.000999  0.000999  0.000999  0.000999  0.000999  0.000999  0.000999  0.000999  0.000999  0.000999  0.000999  0.000999  0.000999  0.000999  0.000999  0.000999  0.000999  0.000999  0.000999  0.000999  0.000999  0.000999  0.000999  0.000999  0.000999  0.000999  0.000999  0.000999  0.000999  0.000999  0.000999  0.000999  0.000999  0.000999  0.000999  0.000999  0.000999  0.000999  0.000999  0.000999  0.000999  0.000999  0.000999  0.000999  0.000999  0.002  0.002  0.002  0.002  0.002  0.002  0.002  0.002  0.002  0.002  0.002  0.002  0.002  0.003  0.003  0.003  0.003  0.003  0.004  0.004  0.004  0.004  0.004  0.004  0.005  0.005  0.005  0.005  0.00599  0.00599  0.00599  0.00699  0.00699  0.00699  0.00699  0.00699  0.00699  0.00699  0.00699  0.00699  0.00699  0.00799  0.00799  0.00899  0.00899  0.00899  0.00999  0.00999  0.011  0.011  0.012  0.013  0.014  0.015  0.015  0.015  0.015  0.016  0.018  0.018  0.018  0.019  0.02  0.02  0.021  0.021  0.024  0.024  0.024  0.026  0.026  0.027  0.029  0.029  0.03  0.03  0.031  0.031  0.033  0.035  0.036  0.036  0.037  0.04  0.04  0.044  0.045  0.045  0.048  0.048 |

**Supplementary Table S2.** Correlation analysis of gut fungal community.

| source | target | weight | correlation |
| --- | --- | --- | --- |
| *Erysiphe* | *Talaromyces*  *Campylospora*  *Russula*  *Pseudaleuria*  *Trichoderma*  *Setophoma*  *Marasmius*  *Purpureocillium*  *Alternaria*  *Humicola*  *Entoloma*  *Plectosphaerella*  *Enterocarpus*  *Thermoascus*  *Chaetomium*  *Didymella*  *Candida*  *Archaeorhizomyces*  *Saitozyma*  *Meyerozyma*  *Vishniacozyma*  *Thelephora*  *Rhodotorula*  *Trichocladium*  *Gibellulopsis*  *Sebacina*  *Debaryomyces*  *Paecilomyces*  *Acremonium*  *Tetracladium*  *Lecanicillium*  *Pseudeurotium*  *Ophiostoma*  *Condenascus*  *Podospora*  *Solicoccozyma*  *Preussia*  *Schizophyllum*  *Fusariella*  *Monascus*  *Trichosporon*  *Cephalotrichum*  *Tausonia*  *Pyrenochaetopsis*  *Cladorrhinum*  *Naganishia*  *Trechispora*  *Staphylotrichum*  *Coniochaeta*  *Oidiodendron*  *Cercospora*  *Filobasidium*  *Zygosaccharomyces*  *Schizothecium*  *Ilyonectria*  *Arxotrichum* | 0.9529  0.8706  0.8735  0.8824  0.7882  0.8471  0.8676  0.8324  0.8176  0.8971  0.85  0.8941  0.6706  0.6529  0.8529  0.8  0.8735  0.6412  0.8971  0.7765  0.8176  0.8029  0.7059  0.6559  0.8706  0.764  0.5618  0.7206  0.7559  0.6412  0.6785  0.65  0.7794  0.7176  0.8971  0.7108  0.6176  0.8529  0.6882  0.8647  0.7647  0.8176  0.6902  0.5765  0.6941  0.5088  0.6941  0.6735  0.7029  0.7618  0.5471  0.7882  0.6896  0.7403  0.5166  0.5618 | positive |
| *Podospora* | *Solicoccozyma*  *Schizophyllum*  *Fusariella*  *Monascus*  *Trichosporon*  *Cephalotrichum*  *Tausonia*  *Pyrenochaetopsis*  *Cladorrhinum*  *Trechispora*  *Staphylotrichum*  *Coniochaeta*  *Oidiodendron*  *Cercospora*  *Filobasidium*  *Zygosaccharomyces*  *Parastagonospora*  *Schizothecium* | 0.7285  0.7588  0.8618  0.7324  0.7588  0.7118  0.8859  0.7176  0.75  0.6882  0.5853  0.6706  0.6588  0.5941  0.8324  0.8031  0.5949  0.6652 | positive |
| *Fusarium* | *Kazachstania*  *Cladosporium*  *Mortierella*  *Metacordyceps*  *Penicillium*  *Botryotrichum*  *Erysiphe*  *Talaromyces*  *Campylospora*  *Russula*  *Pseudaleuria*  *Trichoderma*  *Setophoma*  *Marasmius*  *Purpureocillium*  *Alternaria*  *Humicola*  *Entoloma*  *Plectosphaerella*  *Enterocarpus*  *Thermoascus*  *Chaetomium*  *Didymella*  *Candida*  *Archaeorhizomyces*  *Saitozyma*  *Meyerozyma*  *Vishniacozyma*  *Thelephora*  *Rhodotorula*  *Trichocladium*  *Gibellulopsis*  *Sebacina*  *Debaryomyces*  *Paecilomyces*  *Acremonium*  *Tetracladium*  *Lecanicillium*  *Pseudeurotium*  *Ophiostoma*  *Condenascus*  *Podospora*  *Solicoccozyma*  *Preussia*  *Schizophyllum*  *Fusariella*  *Monascus*  *Trichosporon*  *Cephalotrichum*  *Tausonia*  *Pyrenochaetopsis*  *Cladorrhinum*  *Trechispora*  *Staphylotrichum*  *Epicoccum*  *Coniochaeta*  *Oidiodendron*  *Filobasidium*  *Zygosaccharomyces*  *Leptobacillium*  *Schizothecium*  *Knufia*  *Thanatephorus* | 0.8353  0.9147  0.9794  0.6971  0.8824  0.8882  0.8971  0.8941  0.8382  0.8765  0.8618  0.7059  0.9118  0.8824  0.8059  0.8647  0.8794  0.9176  0.9147  0.7294  0.7029  0.9176  0.8794  0.8529  0.7147  0.8618  0.8441  0.8941  0.7765  0.75  0.7324  0.8559  0.823  0.5176  0.7618  0.7941  0.7647  0.4993  0.5412  0.8294  0.7029  0.8765  0.7403  0.5529  0.7559  0.6912  0.7294  0.8324  0.7676  0.727  0.8029  0.8324  0.8088  0.6059  0.5882  0.7382  0.7353  0.7235  0.6866  0.6206  0.8109  0.5158  0.6342 | positive |
